# Exogenous abscisic acid (ABA) improves the filling process of maize grains at different ear positions by promoting starch accumulation and regulating hormone levels under high planting density

**DOI:** 10.1186/s12870-024-04755-9

**Published:** 2024-01-31

**Authors:** Tao Yu, Yuning Xin, Peng Liu

**Affiliations:** 1 College of Plant Protection, Shandong Agricultural University, Taian, 271018 P.R. China; 2 College of Agronomy, Shandong Agricultural University, Taian, 271018 P.R. China

**Keywords:** Maize, ABA, Grain position, Grain filling, Starch accumulation, Hormone

## Abstract

**Background:**

Higher planting densities typically cause a decline in grain weight, limiting the potential for high maize yield. Additionally, variations in grain filling occur at different positions within the maize ear. Abscisic acid (ABA) is important for grain filling and regulates grain weight. However, the effects of exogenous ABA on the filling process of maize grains at different ear positions under high planting density are poorly understood. In this study, two summer maize hybrids (DengHai605 (DH605) and ZhengDan958 (ZD958)) commonly grown in China were used to examine the effects of ABA application during the flowering stage on grain filling properties, starch accumulation, starch biosynthesis associated enzyme activities, and hormone levels of maize grain (including inferior grain (IG) and superior grain (SG)) under high planting density.

**Results:**

Our results showed that exogenous ABA significantly increased maize yield, primarily owing to a higher grain weight resulting from an accelerated grain filling rate relative to the control. There was no significant difference in yield between DH605 and ZD958 in the control and ABA treatments. Moreover, applying ABA promoted starch accumulation by raising the activities of sucrose synthase, ADP-glucose pyrophosphorylase, granule-bound starch synthases, soluble starch synthase, and starch branching enzyme in grains. It also increased the levels of zeatin riboside, indole-3-acetic acid, and ABA and decreased the level of gibberellin in grains, resulting in more efficient grain filling. Notably, IG exhibited a less efficient filling process compared to SG, probably due to lower starch biosynthesis associated enzyme activities and an imbalance in hormone contents. Nevertheless, IG displayed greater sensitivity to exogenous ABA than SG, suggesting that appropriate cultural measures to improve IG filling may be a viable strategy to further increase maize yield.

**Conclusions:**

According to our results, spraying exogenous ABA could effectively improve grain filling properties, accelerate starch accumulation by increasing relevant enzyme activities, and regulate hormone levels in grains, resulting in higher grain weight and yield of maize under high planting density. Our findings offer more evidence for using exogenous hormones to improve maize yield under high planting density.

## Background

Given the prominence of maize as a staple crop and the escalating global population, it is crucial to make continuous advancements in maize production to ensure worldwide food security [1, 2]. Among the variables contributing to maize yield, grain weight is the most significant determinant after ear number and grains per ear [3]. Among various agronomic practices, increasing density is an excellent way to increase maize yield by optimizing resource utilization [4, 5]. Nevertheless, intense competition for light and nutrients between plants reduces grain weight in high-density environments, limiting the potential to increase maize yield [6, 7]. Previous studies have shown that maize yield follows a quadratic curve with increasing planting density [8, 9]. To further increase maize yield under high planting density, it is therefore essential to use effective agronomic practices to promote plant development and improve grain weight.

During grain development, the rate and duration of filling strongly influence grain weight [10]. Therefore, implementing appropriate agronomic practices can enhance grain weight by accelerating the filling rate or prolonging the filling duration. However, high planting density reduces the grain filling rate and shortens the filling duration, thus reducing maize grain weight [11]. Furthermore, grain development and weight vary significantly at different positions within the maize ear. Generally, grain in the upper part of the ear is known as inferior grain (IG), while that in the middle and lower portions is known as superior grain (SG) [12, 13]. An IG was significantly smaller and weighed less compared to a SG [14]. This distinction is similar in wheat [15] and rice [16]. Inadequate cultivation practices or adverse stresses can further amplify this developmental difference between IG and SG, or even result in IG abortion, ultimately adversely affecting maize yield [17, 18]. Consequently, a great deal of research has gone into finding efficient agronomic techniques to improve IG filling and hence increase crop yield.

Starch, the main storage component of grain, is formed and accumulated during the grain filling process in maize. Maize starch primarily exists in the form of amylose and amylopectin, which are synthesised by key enzymes including sucrose synthase (SuSy), ADP-glucose pyrophosphorylase (AGPase), granule-bound starch synthases (GBSS), soluble starch synthase (SSS), and starch branching enzyme (SBE) [19]. In grains, SuSy converts sucrose to starch, while AGPase determines the starch synthesis rate [20]. GBSS contributes to amylose formation, while SSS and SBE mainly contribute to amylopectin formation [21]. Some research has shown that increasing these enzyme activities through crop cultivation methods like fertilization, irrigation, and chemical control can promote starch accumulation, resulting in higher grain weight [22, 23]. Clearly, variations in these enzyme activities directly affect grain filling and weight by regulating starch accumulation.

Grain development is also closely related to endogenous hormones including cytokinin (ZR), auxin (IAA), abscisic acid (ABA), and gibberellic acid (GA_3_) [24, 25]. These hormones play a crucial role in the morphogenesis and filling of the grain. For instance, the accumulation of ZR and IAA at high levels in grains accelerates the division and growth of endosperm cells, thereby increasing the sink size [26, 27]. ABA and GA_3_ are involved in regulating the accumulation of storage materials in grains by influencing several metabolic enzyme activities [28, 29]. In maize grains, ZR, ABA, and IAA contents correlated positively and significantly with grain filling rate [29]. In wheat [30] and rice [31], SG exhibited significantly higher levels of IAA, ZR, and ABA than IG. Previous studies reported that high planting density increased IAA, ZR, and ABA contents and decreased GA_3_ content in maize grains, thus negatively affecting grain filling [14]. These findings clearly indicate that changes in endogenous hormone levels markedly affect grain filling.

Several reports have demonstrated the usefulness of exogenous hormones in stimulating crop development and increasing yield [32, 33]. Among these hormones, the effects of ABA application on plant development and its regulatory mechanisms have been extensively investigated. For example, the application of ABA increased endogenous hormone contents, enhanced the activities of enzyme for converting sucrose to starch, and improved photosynthetic properties in sweet potato leaves, resulting in higher yield [34]. In wheat, ABA application improved nitrogen metabolism by modulating endogenous hormone contents, thereby promoting protein accumulation in grains [35]. Similarly, ABA application improved the filling process in rice IG by modulating the expression of various proteins associated with basic metabolic pathways [36]. Moreover, ABA application also increased crop tolerance to various adversities, including cold [37], drought [38], salt [39], and heavy metal [40] stress. Yet, the effects of exogenous ABA on grain filling properties, starch accumulation, starch biosynthesis associated enzyme activities, and hormone contents of maize grains under high planting density have received little attention. In particular, the effects of applying ABA on grain filling at various ear positions are poorly understood. Hence, our study investigated the effects of ABA application on the filling process of IG and SG in maize under high planting density, with the aim of providing theoretical and practical insights into the use of exogenous hormones for improving maize yield.

## Results

### Yield, yield components, and economic return

Following ABA treatment, both hybrids showed a significant rise in 1000-grain weight and yield over the control (Table 1). The 1000-grain weight and yield of DH605 showed a significant increase in the ABA treatment, averaging 5.85% and 7.02% over two years relative to the control. Similarly, ZD958 exhibited significant increases of 4.43% and 5.56% in the respective measurements compared to the control. Nevertheless, applying ABA did not significantly affect the ear number and grains per ear in both hybrids. There was no significant difference in yield between DH605 and ZD958 in the control and ABA treatments. In addition, despite increased production costs, exogenous ABA improved the economic return of both hybrids over two years compared to the control.

**Table 1:** Effects of ABA application on yield, yield components, and economic return in 2021 and 2022

Year	Hybrid	Treatment	Ear number (ears hm^− 2^)	Grains per ear	1000-grains weight (g)	Yield (kg hm^− 2^)	Economic return (dollar hm^− 2^)
2021	DH605	CK	84147.36 ± 214 a	434.41 ± 7.63 a	352.73 ± 3.14 c	12893.81 ± 439 b	‒
		ABA	84198.38 ± 523 a	435.37 ± 9.15 a	375.63 ± 4.18 a	13774.32 ± 107 a	268.45 ± 23.75
	ZD958	CK	84143.11 ± 488 a	447.59 ± 6.67 a	346.70 ± 1.77 d	13003.87 ± 237 b	‒
		ABA	84228.38 ± 369 a	445.58 ± 8.91 a	364.58 ± 2.96 b	13669.69 ± 195 a	172.46 ± 36.44
2022	DH605	CK	83893.33 ± 101 a	451.72 ± 17.99 a	357.26 ± 1.03 c	13538.95 ± 263 b	‒
		ABA	83903.47 ± 244 a	460.38 ± 12.36 a	379.48 ± 5.14 a	14514.10 ± 282 a	311.29 ± 22.18
	ZD958	CK	84473.11 ± 581 a	448.98 ± 6.64 a	347.70 ± 9.73 c	13185.40 ± 269 b	‒
		ABA	84948.38 ± 354 a	455.62 ± 9.96 a	361.47 ± 2.04 b	14067.14 ± 175 a	269.29 ± 14.93

CK and ABA mean maize plant in the control and ABA treatments, respectively. Data presented as mean ± S.D. (*n* = 3). Different letters in the same column within the year denote significant differences

### Grain filling process

**Fig. 1:**
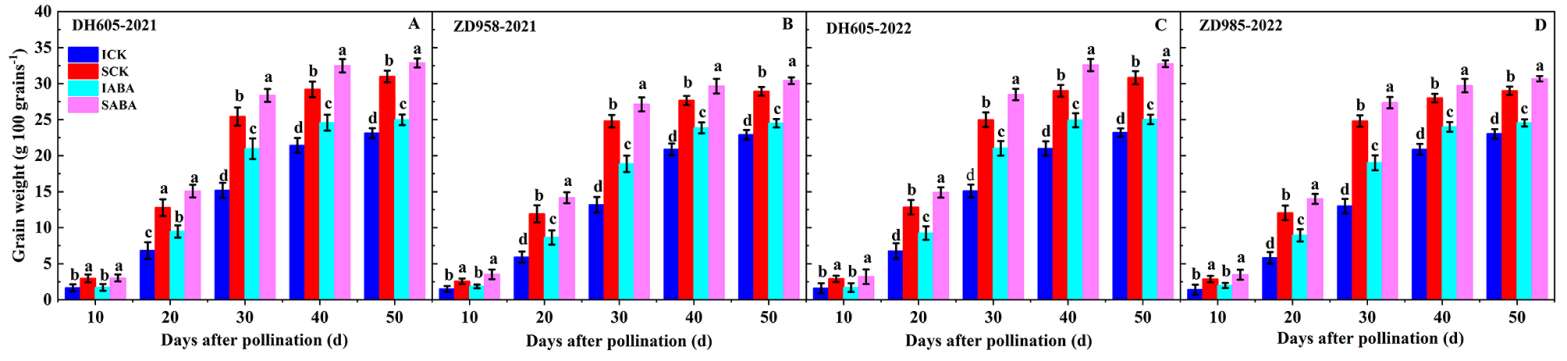
Effects of ABA application on grain dry weight in DH605 (**A** and **C**) and ZD958 (**B** and **D**) during the 2021 and 2022 growing seasons. ICK and IABA mean inferior grain in the control and ABA treatments, respectively. SCK and SABA mean superior grain in the control and ABA treatments, respectively. Means and standard errors are calculated from three replicates. Different letters within a growth period denote significant differences

For both hybrids, IG weighed less than SG at every sampling stage over two years (Fig. 1). Moreover, exogenous ABA increased grain weight in comparison with the control. The increase in IG weight after ABA application was greater than that of SG in both hybrids. At 50 DAP, the two-year mean weight of IG and SG in DH605 after ABA application rose significantly from the control by 7.93% and 6.14%, respectively. Similarly, these values in ZD958 at 50 DAP increased by 6.79% and 5.43%, respectively.

In both hybrids, IG exhibited poorer grain filling properties compared to SG, as evidenced by the significant reduction in grain weight of achieving maximum grain filling rate (W_max_), maximum grain filling rate (G_max_), and average grain filling rate (G_ave_) (Table 2). However, there was no significant difference between IG and SG for active grain filling duration (P). Exogenous ABA markedly improved W_max_, G_max_, and G_ave_, but had no discernible effect on P compared to the control in both hybrids. Moreover, both hybrids exhibited a greater sensitivity of W_max_, G_max_, and G_ave_ to exogenous ABA in IG compared to SG. In DH605, the application of ABA caused a significant increase of 27.63%, 23.65%, and 24.78% in the two-year mean W_max_, G_max_, and G_ave_ in IG, and an increase of 7.77%, 11.70%, and 11.72% in SG over the control. In comparison to the control, for ZD958 treated with ABA, the two-year mean W_max_, G_max_, and G_ave_ of IG were markedly higher by 13.50%, 18.86%, and 20.01%, respectively, while the values of SG were markedly higher by 3.85%, 6.40%, and 5.96%, respectively.

**Table 2:** Effects of ABA application on grain filling properties in 2021 and 2022

Year	Hybrid	Treatment	W_max_ (g 100 grains^− 1^)	G_max_ (g 100 grains^− 1^ d^− 1^)	G_ave_ (g 100 grains^− 1^ d^− 1^)	P (d)
2021	DH605	ICK	11.89 ± 0.41 d	0.92 ± 0.06 d	0.55 ± 0.02 d	38.83 ± 2.57 a
		IABA	15.16 ± 0.60 c	1.12 ± 0.07 c	0.68 ± 0.06 c	41.26 ± 3.68 a
		SCK	17.89 ± 0.40 b	1.35 ± 0.03 b	0.81 ± 0.02 b	39.62 ± 6.00 a
		SABA	18.81 ± 0.44 a	1.45 ± 0.04 a	0.87 ± 0.03 a	39.02 ± 7.90 a
	ZD958	ICK	12.07 ± 0.26 d	0.87 ± 0.04 d	0.52 ± 0.03 d	41.40 ± 4.21 a
		IABA	13.73 ± 0.41 c	1.04 ± 0.05 c	0.63 ± 0.02 c	39.20 ± 6.82 a
		SCK	16.85 ± 0.30 b	1.24 ± 0.04 b	0.75 ± 0.01 b	40.68 ± 5.05 a
		SABA	17.48 ± 0.25 a	1.32 ± 0.03 a	0.79 ± 0.02 a	39.61 ± 8.01 a
2022	DH605	ICK	11.89 ± 0.35 d	0.90 ± 0.04 d	0.54 ± 0.03 d	39.70 ± 3.46 a
		IABA	15.19 ± 0.47 c	1.13 ± 0.06 c	0.68 ± 0.03 c	40.19 ± 4.25 a
		SCK	17.00 ± 0.52 b	1.25 ± 0.07 b	0.75 ± 0.03 b	40.72 ± 6.20 a
		SABA	18.79 ± 0.48 a	1.45 ± 0.06 a	0.87 ± 0.04 a	38.79 ± 8.21 a
	ZD958	ICK	12.16 ± 0.36 d	0.88 ± 0.06 d	0.53 ± 0.05 d	41.47 ± 5.32 a
		IABA	13.77 ± 0.72 c	1.04 ± 0.08 c	0.63 ± 0.03 c	39.61 ± 7.24 a
		SCK	16.90 ± 0.22 b	1.26 ± 0.03 b	0.76 ± 0.02 b	40.11 ± 6.15 a
		SABA	17.57 ± 0.33 a	1.34 ± 0.02 a	0.81 ± 0.02 a	39.19 ± 8.92 a

ICK and IABA mean inferior grain in the control and ABA treatments, respectively. SCK and SABA mean superior grain in the control and ABA treatments, respectively. Data presented as mean ± S.D. (*n* = 3). Different letters in the same column within hybrid in the same year denote significant differences. W_max_, grain weight of reaching maximum grain filling rate; G_max_, maximum grain filling rate; G_ave_, average grain filling rate; P, active grain filling duration

### Starch content

**Fig. 2:**
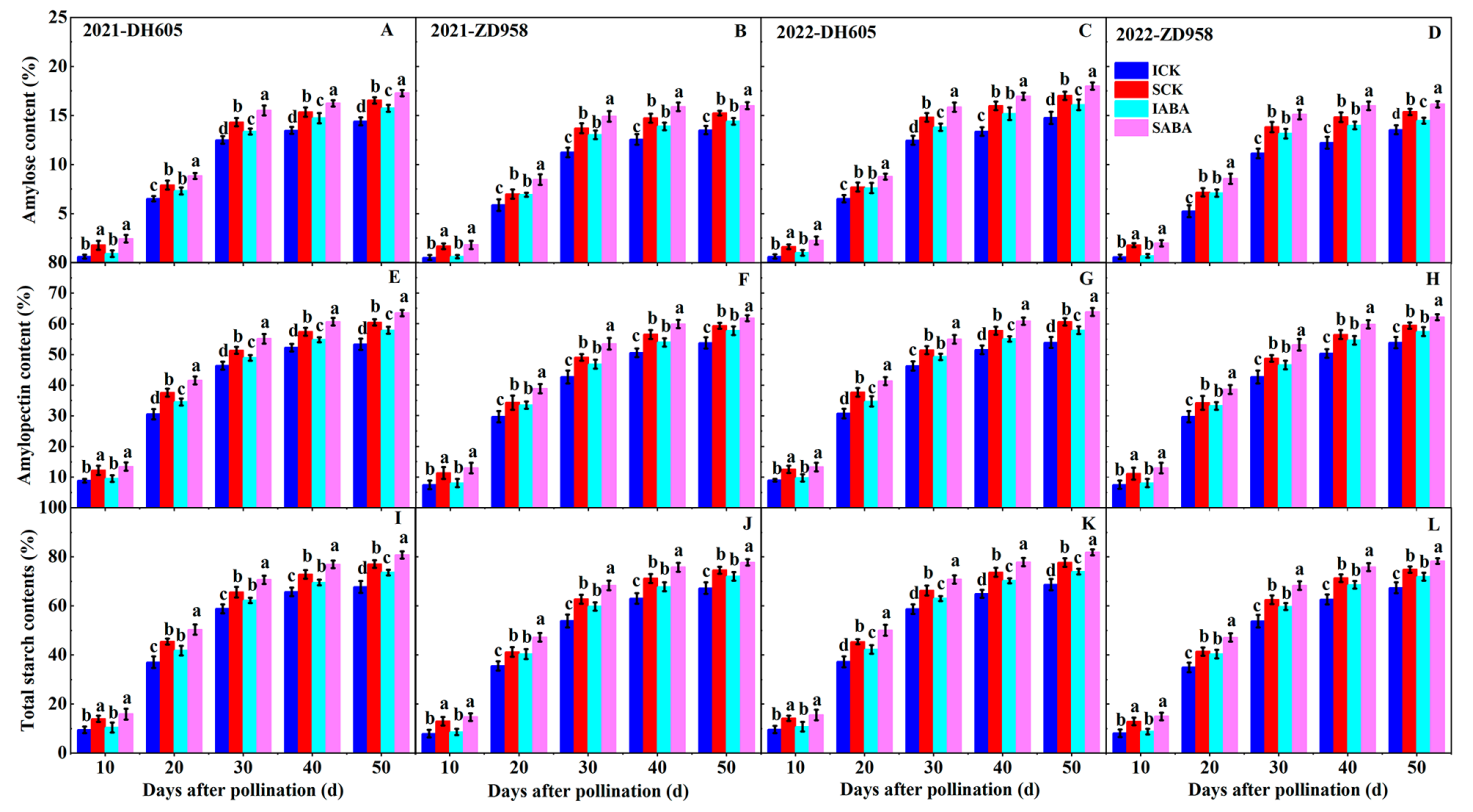
Effects of ABA application on amylose, amylopectin, and total starch contents in DH605 (**A, C, E, G, I and K**) and ZD958 (**B, D, F, H, J and L**) during the 2021 and 2022 growing seasons. ICK and IABA mean inferior grain in the control and ABA treatments, respectively. SCK and SABA mean superior grain in the control and ABA treatments, respectively. Means and standard errors are calculated from three replicates. Different letters within a growth period denote significant differences

For both hybrids, IG contained much less amylose, amylopectin, and total starch contents than SG over two years (Fig. 2). Furthermore, these starch contents improved when treated with ABA over the control, and IG showed higher sensitivity than SG after ABA application. In DH605 at 50 DAP, applying ABA resulted in two-year mean amylose, amylopectin, and total starch contents significantly higher than the control by 9.10%, 7.89%, and 8.15% in IG and by 5.09%, 5.15%, and 5.14% in SG, respectively. In ZD958 at 50 DAP, exogenous ABA significantly improved the two-year mean amylose, amylopectin, and total starch contents by 6.75%, 7.00%, and 6.95% in IG and by 4.98%, 4.35%, and 4.48% in SG, respectively, relative to the control.

### Starch biosynthesis relevant enzyme activities

**Fig. 3:**
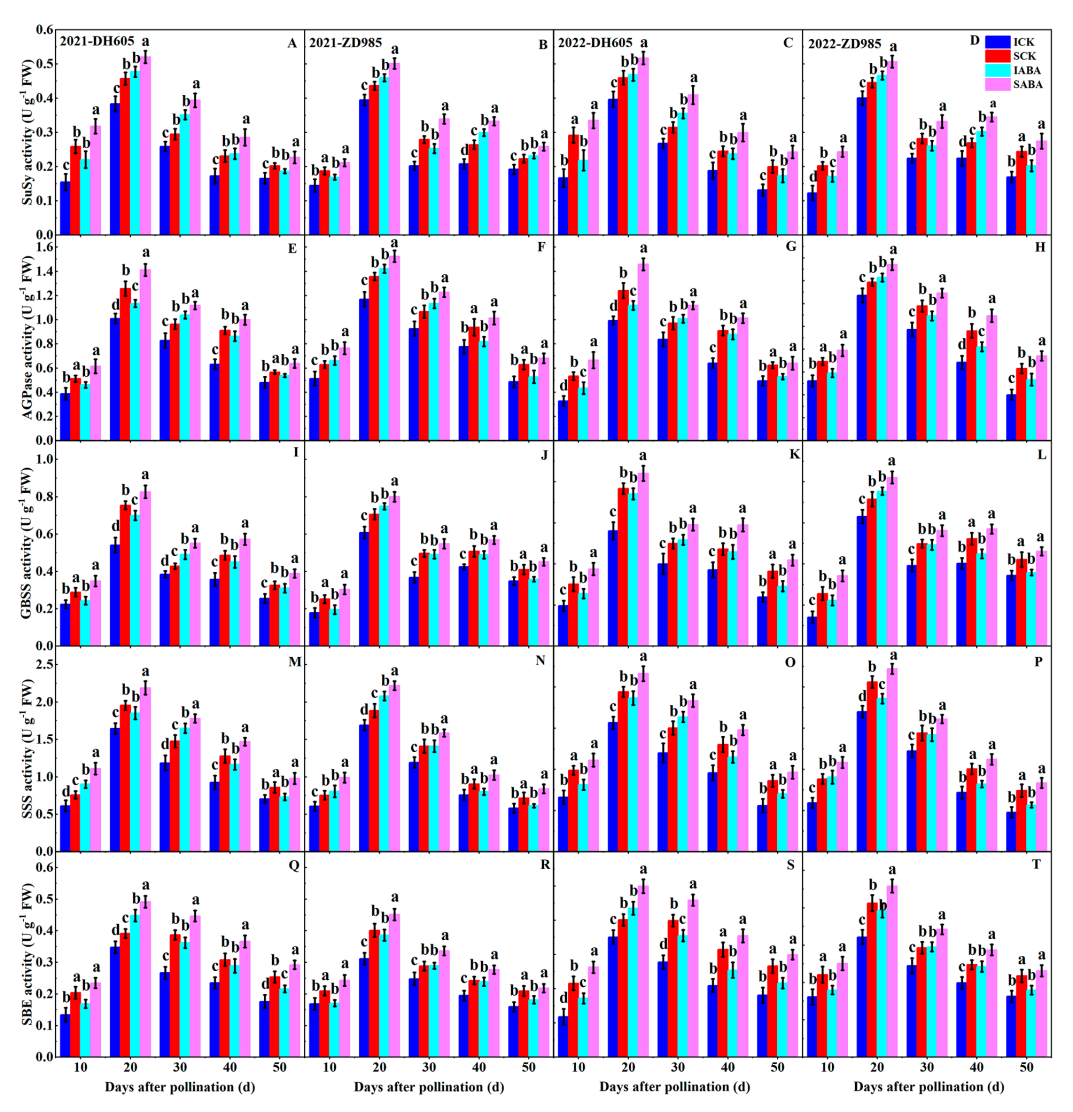
Effects of ABA application on SuSy, AGPase, GBSS, SSS, and SBE activities in DH605 (**A, C, E, G, I, K, M, O, Q and S**) and ZD958 (**B, D, F, H, J, L, N, P, R and T**) during the 2021 and 2022 growing seasons. ICK and IABA mean inferior grain in the control and ABA treatments, respectively. SCK and SABA mean superior grain in the control and ABA treatments, respectively. Means and standard errors are calculated from three replicates. Different letters within a growth period denote significant differences

In both hybrids, SuSy, AGPase, GBSS, SSS, and SBE activities initially rose and then declined from 10 to 50 DAP, with a peak at 20 DAP (Fig. 3). In addition, IG showed lower activities of the above enzyme than SG across every filling stage. Exogenous ABA positively regulated these enzyme activities in both hybrids over the control, with a similar effect observed in both years. Furthermore, these enzyme activities showed a higher sensitivity in IG compared to SG after ABA application. In DH605, applying ABA improved SuSy, AGPase, GBSS, SSS, and SBE activities by 29.18%, 21.56%, 26.50%, 23.24%, and 27.39% in IG and by 20.64%, 14.20%, 20.01%, 15.13%, and 17.65% in SG (average over two years and five sampling periods), respectively, over the control. In ZD958 after ABA application, SuSy, AGPase, GBSS, SSS, and SBE activities of IG were enhanced by 22.94%, 17.74%, 22.07%, 19.45%, and 16.41%, respectively, while those of SG were enhanced by 17.22%, 13.16%, 17.14%, 13.24%, and 12.56% (average over two years and five sampling periods), respectively, relative to the control.

### Grain endogenous hormone levels

**Fig. 4:**
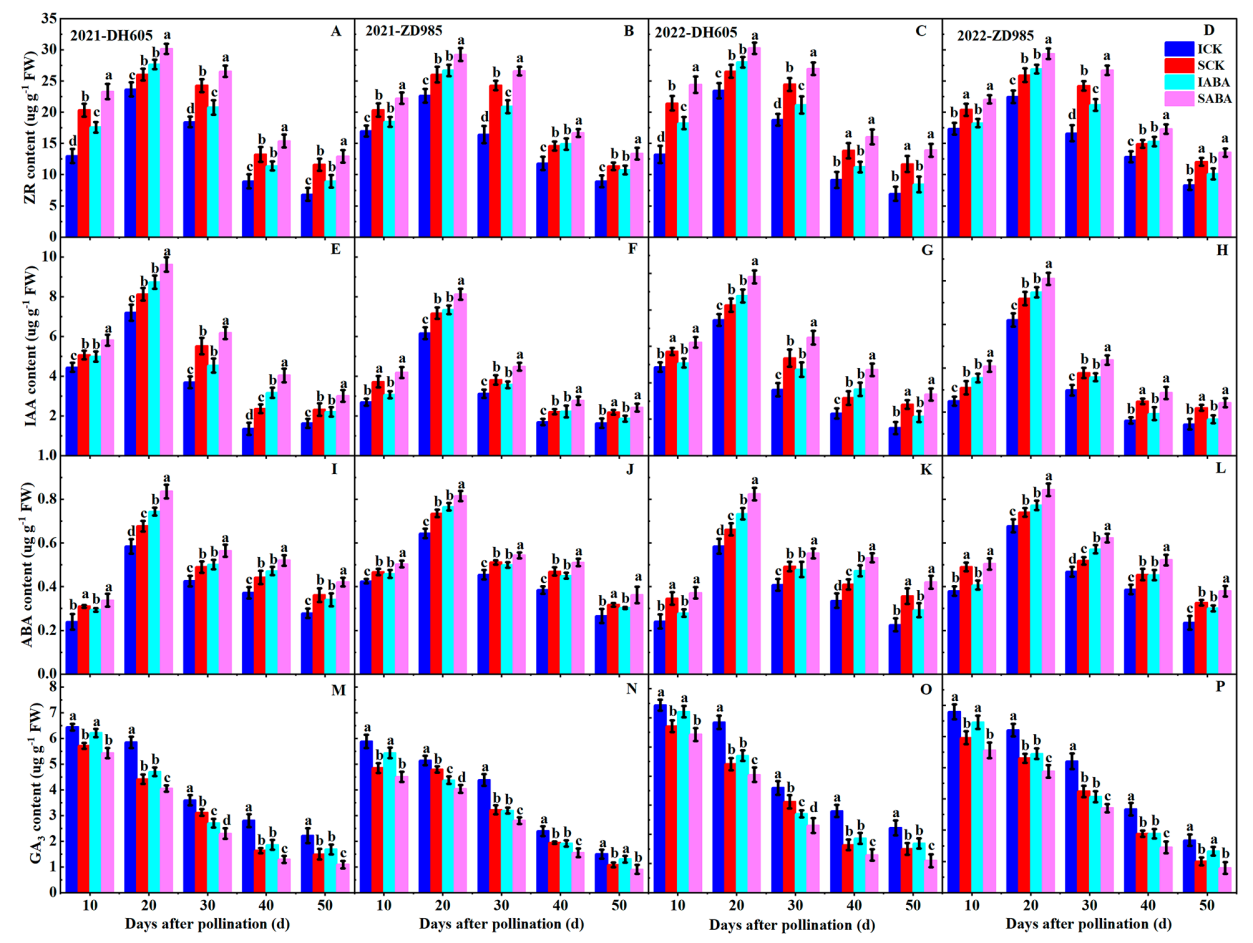
Effects of ABA application on ZR, IAA, ABA, and GA_3_ contents in DH605 (**A, C, E, G, I, K, M and O**) and ZD958 (**B, D, F, H, J, L, N and P**) during the 2021 and 2022 growing seasons. ICK and IABA mean inferior grain in the control and ABA treatments, respectively. SCK and SABA mean superior grain in the control and ABA treatments, respectively. Means and standard errors are calculated from three replicates. Different letters within a growth period denote significant differences

All treatments presented comparable dynamic trends for ZR, IAA, ABA, and GA_3_ levels (Fig. 4). In both hybrids, ZR, IAA, and ABA levels increased from 10 to 20 DAP, peaking at 20 DAP and decreasing thereafter. Conversely, GA_3_ level exhibited a gradual decrease. Additionally, IG showed lower ZR, IAA, and ABA levels than SG in all sampling periods, whereas the opposite was true for GA_3_. The trends of these hormones did not change after ABA treatment. However, applying ABA resulted in increased ZR, IAA, and ABA levels and decreased GA_3_ level compared to the control. This effect was similar in both years. Furthermore, in both hybrids, these hormones showed higher sensitivity to exogenous ABA in IG than in SG. In DH605, ZR, IAA, and ABA levels were increased by 23.16%, 28.43%, and 24.51% in IG and by 14.88%, 21.55%, and 17.26% in SG (average over two years and five sampling periods), respectively, after ABA application relative to the control. In ZD958, exogenous ABA improved ZR, IAA, and ABA levels by 19.23%, 20.90%, and 16.33% in IG and by 12.30%, 16.42%, and 11.74% in SG (average of two years and five sample periods), respectively, over the control. The application of ABA in DH605 resulted in a reduction of GA_3_ level in both IG and SG, by 21.62% and 17.93% (average over two years and five sampling periods), respectively, compared to the control. In ZD958, similar decreases in GA_3_ level of 17.98% and 15.07% (averaged over two years and five sample periods) were observed in IG and SG, respectively.

## Discussion

### Effect of exogenous ABA on grain filling process

Grain weight is highly correlated with the filling process, which is defined by the rate and duration of filling [10]. In our study, SG showed markedly higher maximum and average grain filling rates than IG at high density. The active filling duration did not, however, differ significantly between the two (Table 2). These findings suggest that the slower filling rate may be the primary cause of the inferior filling in IG, resulting in markedly lower grain weight than in SG (Fig. 1). The application of exogenous hormones can regulate the grain development by influencing its filling properties. Applying ABA accelerated grain filling rate and lengthened filling duration, thereby increasing grain weight in wheat [41]. In rice, ABA application shortened grain filling duration but markedly accelerated filling rate, compensating for the negative effect of the shortened filling duration and ultimately resulting in higher grain weight [42]. According to our results, ABA application accelerated the maximum and average grain filling rates in maize grains, while not significantly affecting the active grain filling duration (Table 2). These findings imply that faster grain filling is the primary factor contributing to higher grain weight following ABA application at high density. Interestingly, IG showed higher sensitivity than SG after ABA application in both hybrids, as indicated by the greater increase in grain weight in IG following ABA treatment (Fig. 1). Previous studies have demonstrated that variations in density and nitrogen input have a stronger effect on IG than SG in maize [14]. Soil application of Zn improved maize yield mainly by increasing IG number and weight, but had no such effect on SG [13]. Improper cropping techniques or ecological stressors can hinder the development of IG or even cause it to fail compared to SG in maize [17, 18]. These results imply that IG in maize may be more susceptible to environmental and agronomic changes than SG. Additionally, this effect has also been noted in rice [43] and wheat [15]. As a result, improving IG filling with appropriate agronomic techniques will further improve maize grain yield.

### Effect of exogenous ABA on grain starch accumulation

The starch formation process in maize grains is closely associated with the final grain weight. The ratio and concentration of amylose and amylopectin determine maize starch quality [44]. In our study, IG contained less amylose, amylopectin, and total starch contents compared to SG, further indicating a weaker filling process in IG (Fig. 2). ABA is recognized as a highly effective plant hormone for promoting starch accumulation in plants [45]. Applying ABA altered the distribution of starch granule size and increased starch content in wheat grains [46]. Similarly, this study found that exogenous ABA improved maize grains starch content under close planting conditions, with higher improvement observed in IG than SG (Fig. 2).

SuSy, AGPase, GBSS, SSS, and SBE are all essential for starch accumulation. Our study found that the above enzyme activities exhibited a pattern of increasing and then decreasing during grain development (Fig. 3). Furthermore, our correlation study showed that amylose, amylopectin, and total starch contents were significantly positively correlated with these enzyme activities (Table 3). In agreement with our earlier proteomic findings [47], IG had a decrease in these enzyme activities compared to SG (Fig. 3). This suggests that the lower activities of these enzyme are primarily responsible for the reduced starch content and inefficient filling in IG. In rice grains [48] and in the bioenergy crop duckweed [49], exogenous ABA improved activities of enzyme involved in starch biosynthesis and inhibited activities of enzyme related to starch hydrolysis, thus promoting starch accumulation. Additionally, applying ABA improved starch content by increasing SuSy, AGPase, and SSS activities in maize grains [50]. Similarly, in our current study, exogenous ABA increased SuSy, AGPase, SSS, GBSS, and SBE activities in maize grains (Fig. 3), thereby promoting starch accumulation and increasing starch content (Fig. 2). Notably, these enzyme activities exhibited greater sensitivity to ABA application in IG compared to SG, possibly explaining the greater increase in starch content and grain weight observed in IG after ABA treatment. Earlier studies have suggested that exogenous hormones may regulate the enzyme activities involved in starch accumulation by affecting the expression of relevant genes or proteins [51, 52]. However, the mechanisms by which exogenous ABA modulates the activities of enzyme relevant to starch accumulation in maize grains at various ear positions require further investigation. In summary, our findings suggest that exogenous ABA can enhance starch accumulation in maize grains under high planting density by increasing relevant enzyme activities, thereby improving grain filling.

**Table 3:** Correlation coefficients of starch content with starch biosynthesis-related enzyme activities

Year	Starch content	SuSy	AGPase	GBSS	SSS	SBE
2021	Amylose	0.923^**^	0.784^*^	0.880^**^	0.960^**^	0.964^**^
	Amylopectin	0.940^**^	0.760^*^	0.926^**^	0.950^**^	0.947^**^
	Total starch	0.937^**^	0.743^*^	0.916^**^	0.954^**^	0.952^**^
2022	Amylose	0.948^**^	0.806^*^	0.896^**^	0.985^**^	0.973^**^
	Amylopectin	0.974^**^	0.851^**^	0.946^**^	0.991^**^	0.973^**^
	Total starch	0.970^**^	0.841^**^	0.936^**^	0.992^**^	0.976^**^

SuSy, sucrose synthase; AGPase, ADP-glucose pyrophosphorylase; GBSS, granule-bound starch synthase; SSS, soluble starch synthase; SBE, starch branching enzyme. ^*^ and ^**^ denote significant level of 0.05 and 0.01 (*n* = 8), respectively

### Effect of exogenous ABA on grain endogenous hormone levels

Grain development is closely associated with endogenous plant hormones. Our correlation study revealed a significant positive relationship between the maximum and mean grain filling rates and ZR, IAA, and ABA contents, but a significant negative relationship with GA_3_ content (Table 4), similar to previous research [14]. Differences in the endogenous hormone contents of IG and SG have been proposed as a crucial factor influencing the filling process in wheat [30] and rice [31]. Compared to SG, IG had significantly lower ZR, IAA, and ABA contents in both hybrids in our study (Fig. 4). The significant accumulation of ZR and IAA in early grain filling can enhance grain sink size and strength by accelerating endosperm cell division and development [43]. Additionally, high ABA content in grains can accelerate the grain filling rate by facilitating the translocation of carbohydrates into the grain [42]. Therefore, poorer sink size and strength in IG compared to SG may be caused by lower ZR, IAA and ABA contents, resulting in inefficient filling and lower grain weight in IG. Conversely, IG had a significantly higher GA_3_ content than SG (Fig. 4), similar to a previous report [14]. However, high GA_3_ content in grains could accelerate starch hydrolysis by enhancing α-amylase and other hydrolase activities, ultimately negatively affecting starch accumulation [53, 54].

**Table 4:** Correlation coefficients of endogenous hormone contents with grain filling parameters

Year	Hormone contents	W_max_	G_max_	G_ave_	P
2021	ZR	0.757^*^	0.759^*^	0.757^*^	-0.176
	IAA	0.728^*^	0.761^*^	0.755^*^	-0.376
	ABA	0.783^*^	0.778^*^	0.779^*^	-0.141
	GA_3_	-0.801^*^	-0.792^*^	-0.791^*^	0.123
2022	ZR	0.802^*^	0.832^*^	0.839^**^	-0.679
	IAA	0.733^*^	0.755^*^	0.745^*^	-0.573
	ABA	0.771^*^	0.789^*^	0.800^*^	-0.547
	GA_3_	-0.818^*^	-0.831^*^	-0.840^**^	0.562

ZR, zeatin riboside; IAA, indole-3-acetic acid; ABA, abscisic acid; GA_3_, gibberellin; W_max_, grain weight of reaching maximum grain filling rate; G_max_, maximum grain filling rate; G_ave_, average grain filling rate; P, active grain filling duration. ^*^ and ^**^ denote significant level of 0.05 and 0.01 (*n* = 8), respectively

Various investigations have revealed that several hormones rather than a single hormone are responsible for modulating grain filling in response to exogenous hormones [32]. The application of ABA to rice raised ABA content and reduced GA_3_ content in grains, thereby improving grain filling [55]. In wheat grains, applying ABA also improved grain filling properties and promoted starch formation by raising ZR, IAA, and ABA contents and reducing GA_3_ content [41]. According to our results, exogenous ABA raised IAA, ZR, and ABA contents while reducing GA_3_ content in maize grains (Fig. 4). These alterations in hormone content could enhance the grain sink capacity and strength, which would accelerate filling rate and carbohydrate accumulation, thus improving the filling process and increasing grain weight [41, 55]. Previous studies reported that fluctuations in endogenous hormone levels in IG of maize exhibited higher sensitivity to variations in density and nitrogen input than in SG [14]. In our study, exogenous ABA resulted in a more robust modulation of ZR, IAA, ABA, and GA_3_ contents in IG than in SG (Fig. 4). This difference may be a crucial factor contributing to the superior filling performance of IG after ABA application. It has been shown that exogenous hormone can influence endogenous hormone levels through their effect on the regulation of genes or enzymes responsible for hormone biosynthesis, metabolism, and signaling [32]. Thus, it is crucial to further investigate the regulatory mechanisms of endogenous hormone dynamics in maize grains from various ear positions under high planting density after ABA application. Apparently, ABA application can also promote grain filling by modulating the endogenous hormone contents.

### Effect of exogenous ABA on grain yield under high planting density

Exogenous hormones are widely employed in field management techniques as an effective method of increasing crop yield [32]. Applying ABA could enhance the leaf photosynthetic capacity, spikelet source-to-sink capacity, and pollen viability, thereby increasing rice grain yield [39]. Additionally, the combined application of ABA and sucrose increased grain weight and yield in rice by effectively coordinating the source-sink relationship [28]. In wheat, ABA application raised grain weight and yield by delaying leaf senescence and promoting dry matter translocation into the grain [30, 55]. This study demonstrated that exogenous ABA significantly improved maize 1000-grain weight and yield over the control without significantly affecting ear number and grains per ear (Table 1), suggesting that exogenous ABA increases maize yield primarily by increasing grain weight under high planting density. Importantly, the increase in grain weight could be attributed to exogenous ABA improving filling properties, stimulating starch biosynthesis by increasing relevant enzyme activities, and modifying endogenous hormone levels in grains. It should be noted that only two maize hybrids were used in this study to assess the effect of ABA application on grain filling and yield. It is therefore crucial to further investigate the yield advantage data with a larger number of varieties, which would have strengthened the practical utility and general applicability. Our results also showed that applying ABA increased the economic return of maize despite increased production costs (Table 1), further suggesting that this agronomic practice is worthy of widespread promotion and application in maize production.

## Conclusions

Under high planting density, IG exhibited less efficient filling than SG, probably due to a reduction in starch biosynthesis-related enzyme activities and an imbalance in endogenous hormone levels. As a result, IG had markedly lower starch content and grain weight compared to SG. Exogenous ABA was effective in improving grain filling properties, accelerating starch accumulation by increasing relevant enzyme activities, and regulating endogenous hormone levels in grains, thus contributing to adequate grain filling and ultimately higher maize grain weight and yield under high planting density. In particular, IG exhibited higher sensitivity to exogenous ABA than SG, suggesting that appropriate cultural measures to improve IG filling may be a viable strategy to further increase maize yield.

### Methods

#### Trial site

**Fig. 5:**
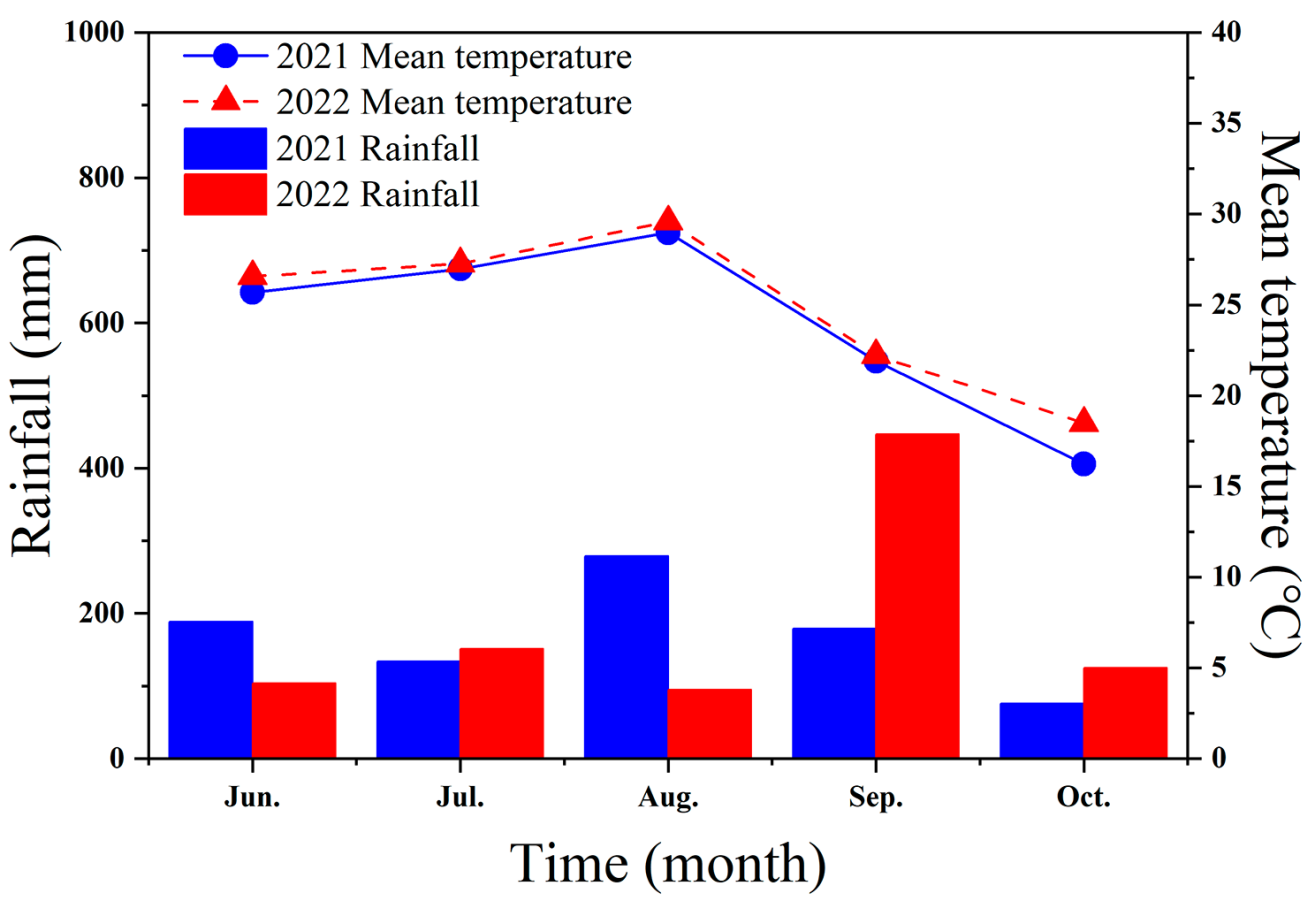
Data on the mean rainfall and temperatures for the maize cropping period in 2021 and 2022

The trial took place in 2021 and 2022 on the Shandong Agricultural University farm in China (36°10′N, 117°04′E). The mean temperature and rainfall statistics for the maize growth seasons of the two years are shown in Fig. 5. The test region is defined by its brown loam soil, and the organic matter, alkali-hydrolysable nitrogen, available phosphorus, and available potassium contents in the top 20 cm of soil were 12.36 g kg^− 1^, 55.43 mg kg^− 1^, 45.79 mg kg^− 1^, and 78.89 mg kg^− 1^, respectively.

#### Trial design and sampling

Two summer maize hybrids commonly cultivated in China, Denghai 605 (DH605) and Zhengdan 958 (ZD958), were selected for the experimental materials. Seeds of both hybrids were supplied by China National Seed Group CO., LTD., Beijing, China. Both hybrids were planted on June 14th at a high density (90,000 plants ha^− 2^). During the flowering stage, ABA was uniformly sprayed on all maize leaves using a sprayer, while the control received a water spray. The ABA and water spraying occurred from 16:00 to 18:00 for three consecutive days. A dose of 0.5 mmol L^− 1^ ABA was used, based on previous research [56]. ABA was applied at 150 ± 5 ml per plant. Each treatment was repeated in triplicate using a fully randomized design. Every plot consisted of five rows, spaced 60 cm apart, resulting in a total area of 12 m by 3 m. Regarding fertilization, every plot received 280 kg ha^− 1^ N, 100 ha^− 1^ P_2_O_5_, and 220 kg ha^− 1^ K_2_O. Phosphorus, potassium, and 50% of the nitrogen fertilizer were spread before sowing, and the remaining nitrogen fertilizer was spread during the jointing period. Irrigation, weed control, disease management, pest control, and other necessary management practices were maintained uniformly across all treatments.

At the tasseling period, a minimum of 150 robust and evenly developing plants were chosen for each plot. Artificial pollination was used to ensure consistency of pollination. Subsequently, five marked plants were chosen from each plot, and their ears were sampled every 10 days from 10 to 50 days after pollination (DAP). After that, the ears were separated into top, middle, and bottom regions. The middle and top grains were labelled SG and IG, respectively. Half of the grains were frozen in liquid nitrogen and kept at -80 °C to examine starch biosynthesis-related enzyme activities and hormone levels. The other grains were dried at 80 °C to obtain a consistent weight after baking at 105 °C for 30 min to evaluate the grain filling properties and starch content.

### Grain filling dynamics

Grain dry weight was calculated from hundred IG and SG. The logistic equation (y = A / (1 + Be ^− Ct^)) proposed by [57] was adopted to match the filling process. After that, filling parameters were calculated, including the grain weight of achieving maximum grain filling rate (W_max_ = A / 2), the maximum grain filling rate (G_max_ = (C × W_max_) × [1 − (W_max_ / A)]), the average grain filling rate (G_ave_ = (95% of A − 5% of A) / (t_2_ − t_1_)), and the active grain filling duration (*P* = 6 / C). The variables in the equation are the grain weight (y), the final grain weight (A), the days after pollination (t), and the regression coefficients (B and C). The days when 5% and 95% of A are completed are called t_1_ and t_2_, respectively.

### Measurement of starch content and starch biosynthesis relevant enzyme activities

The “double wavelength” method proposed by [58] was utilized to quantify amylose and amylopectin contents. Two different wavelengths were employed for this purpose: 556 and 737 nm for measuring amylose content, and 620 nm and 479 nm for assessing amylopectin content. Total starch content was calculated as the sum of amylose and amylopectin. SuSy, AGPase, GBSS, SSS, and SBE activities were determined with the corresponding assay kits (Suzhou Comin Biotechnology Co., Ltd., Suzhou, China). Three biological replicates were used to determine these indicators.

### Measurement of endogenous hormone levels

ZR, IAA, ABA, and GA_3_ levels were determined by high-performance liquid chromatography as recommended by [59]. Each hormone was measured against standards supplied by Suzhou Comin Biotechnology Co., Ltd., Suzhou, China. The calibration curve for each hormone was built with standards that ranged from 0 to 0.2 mg mL^− 1^. Three biological replicates were used to determine these indicators.

### Measurement of yield, yield components, and economic return

At physiological maturity, 30 ears were harvested from the central area of each plot to investigate yield (moisture content was 14%), grains per ear, and 1000-grain weight. We also assessed whether the ABA treatment could increase the economic return of maize compared to the control treatment. Economic return = (yield in ABA treatment − yield in control treatment) × maize price − ABA cost. The maize price was 0.45 dollar kg^− 1^ and the ABA cost was 126.12 dollar hm^− 2^. The data needed to determine the economic return was obtained by surveying local farms during the two years studied.

### Statistical analysis

Statistical analyses were made with SPSS 28.0 (SPSS Inc., Chicago, IL, USA). Significance differences between treatments were determined using Duncan’s test (*p* = 0.05). SigmaPlot 12.0 was used to create the figures.

## Data Availability

All data generated during this study are included in this published article.

## Abbreviations

ABA: Abscisic acid
AGPase: ADP-glucose pyrophosphorylase
DAP: Days after pollination
DH605: Denghai 605
GA_3_: Gibberellin
G_ave_: Average grain filling rate
GBSS: Granule-bound starch synthases
G_max_: Maximum grain filling rate
IAA: Indole-3-acetic acid
IG: Inferior grain
P: Active grain filling duration
SBE: Starch branching enzyme
SG: Superior grain
SSS: Soluble starch synthase
SuSy: Sucrose synthase
W_max_: Grain weight of reaching maximum grain filling rate
ZD958: Zhengdan 958
ZR: Zeatin riboside
